# Fluorite material sources and magmatic–tectonic controls in hydrothermal fluorite deposits

**DOI:** 10.1371/journal.pone.0353161

**Published:** 2026-07-20

**Authors:** Fengyu Sun, Changling Qu, Xin Du, Xiujun Zhao, Yao Dong, Gaoshe Cao, Xinhang Zhou, Xiaochen Ma

**Affiliations:** 1 Institute of Resources and Environment, Henan Polytechnic University, Jiaozuo, Henan, China; 2 Henan Geological Bureau, Zhengzhou, Henan, China; 3 Henan Key Laboratory of Coal Measure Unconventional Resources Accumulation and Exploitation, Jiaozuo, Henan, China; Maria Curie-Sklodowska University: Uniwersytet Marii Curie-Sklodowskiej, POLAND

## Abstract

A fundamental yet unresolved issue in hydrothermal fluorite deposits is that initial ^143^Nd/^144^Nd ratios vary significantly among different veins, even among those with similar mineralization ages within the same region. This discrepancy likely reflects variations in the material sources of hydrothermal fluids, although the specific controlling factors and mechanisms remain unclear. To address this issue, this study analyses two types of fluorite veins on the southern margin of the North China Craton—both controlled by the same fault system and the same pluton—using Sm-Nd and C-O isotopes. The Baimiaogou fluorite veinlets represent conduit structures for hydrothermal migration, whereas the Yangshan fluorite veins represent host structures for ore precipitation. The results show that the Baimiaogou veinlets, which acted as fluid conduits, display highly variable isotopic compositions, with initial ^143^Nd/^144^Nd ratios of 0.511379 to 0.511757, *ε*_Nd_(*t*) values of –21.55 to –14.17, and *T*_DM2_ ages of 1.64 to 1.93 Ga. Associated calcite exhibits a pronounced oxygen isotope shift, with *δ*^18^O_**V-SMOW**_**‰** values between 1.6‰ and 7.0‰. In contrast, the Yangshan veins, which served as host structures, show isotopic consistency, with tightly clustered initial ^143^Nd/^144^Nd ratios (0.511751–0.511799), *ε*_Nd_(*t*) values (–14.29 to –13.36), *T*_DM2_ ages (1.58–1.60 Ga), and associated calcite *δ*^18^O_**V-SMOW**_**‰** values (7.5–8.5‰). Neodymium isotope mass balance calculations indicate that the variations in Nd isotope values reflect mixing between initial magmatic-hydrothermal fluids and the ancient Taihua Group metamorphic basement during fluid migration. Macro- and microstructural analyses reveal that the Baimiaogou veinlets experienced multiple tectonic pulses within an open system characterised by dynamically changing fluid flow paths, whereas the Yangshan veins formed during a single phase of large-scale extensional faulting, with fluid precipitation occurring in a relatively closed space. Thus, the nature and pulsatory behaviour of fault structures also control the variations in Nd isotope values. This model highlights the coupled control of magmatism and structural processes on the material sources of fluorite in hydrothermal deposits, providing new constraints for understanding the metallogenic mechanisms of hydrothermal fluorite systems.

## Introduction

The southern margin of the North China Craton hosts a significant fluorite (CaF_2_) metallogenic belt in China, where numerous hydrothermal fluorite deposits (veins) of magmatic-structural affinity are present [1–3]. A long-standing focus in understanding the genesis of these fluorite deposits has been their material sources. Previous studies, utilizing rare earth element (REE) patterns of fluorite and comparisons with regional ore-hosting granites and stratigraphic units, suggest that the ore-forming materials in this region are derived mainly from granites [4–7] or from the host strata [8–12], and that magmatic-hydrothermal fluid-wall rock interactions controlled mineralization. However, using REE characteristics to trace ore-fluid sources has certain limitations. Rare earth elements readily fractionate during fluid migration and fluid-rock interactions, meaning that their distribution patterns often do not accurately preserve the geochemical signature of the primary ore-forming fluid, leading to ambiguous interpretations [13–22]. In contrast, the Sm-Nd isotope system is more stable within the fluorite lattice and can effectively resist later hydrothermal disturbance; moreover, the initial ^143^Nd/^144^Nd ratio of fluorite directly reflects the material source and evolution path of the ore-forming fluid [23–27].

Despite extensive previous Sm-Nd chronological studies on hydrothermal fluorite deposits, a critical paradox has been widely overlooked: initial ^143^Nd/^144^Nd ratios exhibit pronounced variations among different ore veins (or deposits) within the same metallogenic belt but remain remarkably consistent within a single deposit or vein [6,28–34]. A systematic comparative analysis and discussion regarding the factors controlling these isotopic signatures are lacking.

This study focuses on the fluorite veinlets (width <20 cm) in the Baimiaogou area and the major fluorite veins (width >1 m) in the Yangshan area, both of which are controlled by NW-trending faults along the southern margin of the North China Craton. By combining combined Sm-Nd and C-O isotope analysesand integrating them with an investigation of magmatism, structural features, and regional fluorite vein comparisons, the study aims to (1) determine the material sources and elucidate the discrepancies between these two types of veins and (2) decipher the controls of magmatic-tectonic processes on fluid isotopic tracing. These findings provide new geochemical constraints for better understanding the genetic mechanisms of hydrothermal vein-type fluorite deposits.

## Regional geology

**Fig 1:**
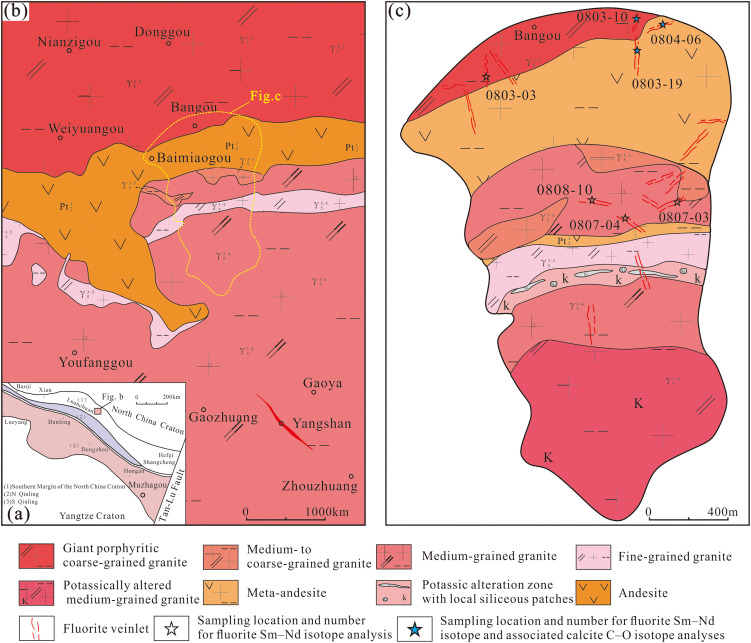
Simplified tectonic framework and geological maps of the Baimiaogou area in the western Henan region, southern margin of the North China Craton. (a)-Tectonic setting of the study area; (b)-Simplified geological map of the study area; (c)-Geological map of the Baimiaogou fluorite veinlets; The Figs a, b, and c were created based on base maps sourced from Natural Earth (http://www.naturalearthdata.com/), which were further processed using software CorelDRAW X8.

The study area is located in the western Henan region along the southern margin of the North China Craton (Fig 1a and 1b), bounded by the Luanchuan fault to the south. The regional stratigraphy, from bottom to top, consists of the Late Archean to Palaeoproterozoic Taihua Group (basement), the Mesoproterozoic Xiong’er Group volcanic rocks, and the Meso- to Neoproterozoic Guandaokou and Luanchuan groups (cover sequences) (Fig 1c).

Regional fault structures are well developed, among which the NW-trending Machaoying and Luanchuan faults are the most significant. Extending for more than 200 Km, the Machaoying fault experienced multistage activity and was reactivated in the Early Cretaceous, serving as the principal ore-controlling structure for fluorite veins in the region (Fig 1a). The large-scale intrusion of Late Jurassic to Early Cretaceous granitic batholiths is a product of lithospheric thinning and extension in the North China Craton. The Heyu composite batholith, the largest Mesozoic pluton in the area, exhibits high-fluorine geochemical characteristics, providing the material source for regional fluorite mineralization [35–40].

The host rock of the Baimiaogou fluorite veinlets is Heyu granite, which can be divided into porphyritic coarse-grained monzogranite, medium-grained monzogranite, and fine-grained monzogranite on the basis of grain size. Controlled by the NW-trending fault zone, the fluorite veinlets are hosted in dense subvertical (dip angles of 70–87°) fracture swarms striking NW, NE, and nearly EW. These fractures are filled with fluorite of varying colours (e.g., smoky white, light green, dark blue, and blue-purple) and degrees of crystallization. The veinlets are generally <20 cm in width and occur either as solitary veins or as swarms of 3–4 veinlets (Fig 1c).

The Yangshan fluorite vein is an economically significant, large-scale fluorite deposit in the region. Within the tectonic fracture zone of the medium- to fine-grained Heyu granite, sharp contact with the wall rocks occurs. The vein strikes NW (generally 295°–310°) with a dip of 70°–80°, and displays a gently undulating geometry along both its strike and dip (Fig 1d). The ore bodies are primarily localised in areas with local variations in structural attitude, occurring in lenticular or pod-like forms. Individual ore bodies have a strike length exceeding 400 m and a general thickness of 1–3 m, with the maximum thickness reaching more than30 m. The fluorite is predominantly blue and brown.

## Sample collection and analytical methods

Sample CollectionIn this study, representative fluorite samples exhibiting various modes of occurrence, colours, crystallinities, and textural-structural characteristics were systematically collected from the Baimiaogou fluorite veinlets and the Yangshan fluorite veins for Sm-Nd isotope analysis. Additionally, calcite paragenetically associated with fluorite in selected samples was analysed for C-O isotopes.

**Fig 2:**
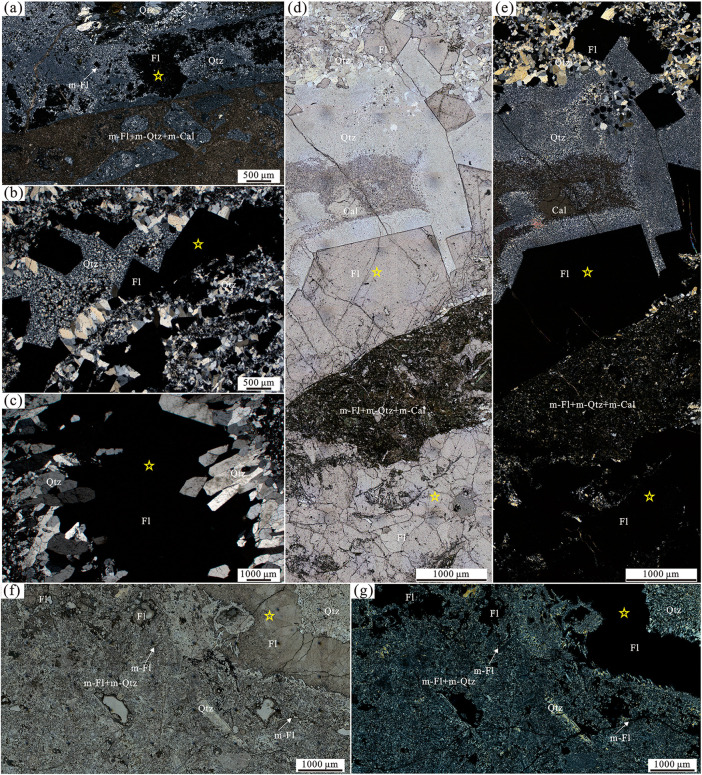
Photomicrographs of samples from the Baimiaogou fluorite veinlets and the Yangshan fluorite veins in the Yuxi region, southern margin of the North China Craton. Yellow stars indicate sampling locations for Sm-Nd isotope analysis. (a) Photomicrograph of a fluorite veinlet (attitude: 181°∠85°). Poorly crystallised fluorite contains fine-grained calcite and quartz inclusions, whereas well-crystallised fluorite infills the interstices of comb-like quartz (Sample No. 0803−03); (b) Photomicrograph of a fluorite veinlet (attitude: 111°∠76°). subhedral to euhedral quadrangular fluorite crosscuts quartz grains and comb-like quartz (Sample No. 0807−03); (c) Photomicrograph of a fluorite veinlet (attitude: 151°∠90°). well-crystallised fluorite occurs as vein-like infillings within the interstices of comb-like quartz (Sample No. 0803−19); (d) Photomicrograph of a fluorite veinlet (attitude: 11°∠84°). euhedral quadrangular fluorite crystals crosscut quartz grains and comb-like quartz (Sample No. 0807−04); (e) cross-polarised light image of the same view as (d); (f) photomicrograph of the Yangshan fluorite vein (attitude: 213°∠75°). Well-crystallised fluorite forms euhedral quadrangular or hexagonal crystals, with crystal edges crosscutting comb-like quartz grains or infilling their interstices and poorly crystallised fluorite is disseminated interstitially among microcrystalline quartz grains (Sample No. 240316−01); (g) cross-polarised light image of the same view as (f). Mineral abbreviations: Fl = fluorite; Qtz = quartz; Cal = calcite; m-Fl = cryptocrystalline fluorite; m-Qtz = microcrystalline quartz.

For the Baimiaogou deposit, samples were collected from fluorite veinlets with different occurrences within the fault zone (Fig 2a–2c), with certain samples taken from different positions along a single veinlet (Fig 2d and 2e). Petrographic observations reveal that fluorite is intergrown with quartz and calcite in the veinlets, resulting invarying crystallization characteristics. Well-crystallised fluorite occurs as hexagonal or irregular polygonal crystals, whose crystal edges crosscut calcite and quartz (Fig 2). In contrast, poorly crystallised, cryptocrystalline fluorite occurs in vein-like form and frequently contains fine-grained calcite and quartz inclusions (Fig 2a). Quartz is present as microcrystalline aggregates and has a comb-like texture (Fig 2a–2c). Calcite occurs mainly as irregular patches or fine-grained relics preserved within cryptocrystalline fluorite veinlets and microcrystalline quartz aggregates (Fig 2a, 2d and 2e).

Samples from the Yangshan fluorite veins were collected from a single, thick orebody, where the fluorite is predominantly brown, blue-green, and variegated in colour. Petrographic observations indicate that the fluorite displays varying degrees of crystallinity. Well-crystallised, euhedral fluorite forms quadrangular or hexagonal crystals, whose edges typically crosscut comb-like quartz grains or infill the interstices between them. Poorly crystallised fluorite is anhedral, fine-grained, and disseminated interstitially among the microcrystalline quartz grains (Fig 2f and 2g).

The Fig 2 was captured by the author via microscopy photography of actual collected samples; no copyrighted material was used.

### Analytical methods

Sm-Nd isotope analyses were performed at the Isotope Laboratory (Tianjin Center) of the China Geological Survey.The detailed analytical methodology for fluorite Sm-Nd isotopes is described in reference [41,42], and the procedure is briefly outlined below:Sample processing: Single mineral grains with a purity greater than 99% were handpicked under a binocular microscope, cleaned sequentially with ultrapure water and ethanol, dried at low temperatures, and then ground to less than 200 mesh.Separation and purification: The powder samples were repeatedly rinsed and soaked in ultrapure water, ethanol, and 0.5 mol/L acetic acid, and subsequently digested using aqua regia and H_3_BO_3_. Rare earth elements (REEs) were separated from the digested solution using cation exchange resins. The obtained solution was evaporated to dryness and redissolved in HCl and the Sm and Nd fractions were further separated and purified through ion exchange chromatography.Instrumental analysis: The Sm and Nd isotopic compositions were determined using a Thermo Scientific Triton thermal ionization mass spectrometer (TIMS). All sample preparation and analytical procedures were conducted in an ultraclean laboratory. The international rock reference material BCR-2 (basalt) was used to monitor the chemical separation and analytical precision (the detailed parameters are listed in S1 Table). Mass fractionations of Nd isotopes were normalised to ^146^Nd/^144^Nd = 0.7219. The measured ^143^Nd/^144^Nd ratio for the Jndi-1 isotopic standard was 0.512110 ± 5. Isochron fitting and age calculations were performed using the Isoplot software package.

C-O isotopic analyses were completed at the Key Laboratory of Metallogeny and Mineral Assessment, Ministry of Land and Resources, Institute of Mineral Resources, Chinese Academy of Geological Sciences. The measurements were performed using the GasBench II continuous-flow system coupled with a MAT 253 mass spectrometer. The samples were crushed to 200 mesh, placed in reaction vials, and flushed with high-purity helium. Anhydrous phosphoric acid was then added, and the mixture was reacted and equilibrated on a heating tray at 72 °C. The evolved gas was passed through a 70 °C fused silica capillary column to separate the impurities, and the isotopic compositions were subsequently determined using a MAT 253 mass spectrometer.*δ*^13^Cvalues are reported relative to the V-PDB standard, and*δ*^18^Ovalues are reported relative to both the V-PDB and V-SMOW standards, with an analytical precision of ±0.1‰.

## Results

### Sm-Nd isotopes

The results of fluorite Sm-Nd isotope analyses for the Baimiaogou veinlets and Yangshan veins are compiled in S1 Table. The Sm contents of fluorite veinlets in the Baimiaogou deposit range from 0.71 to 4.873 ppm; the Nd contents range from 3.85 to 15.67 ppm, the Sm/Nd ratio varies from 0.16 to 0.443, with an average of 0.223; the ^147^Sm/^144^Nd ratios range from 0.0964 to 0.2679, with an average of 0.15643; and the ^143^Nd/^144^Nd ratios range from 0.511474 to 0.511876, with an average of 0.511677. The Sm concentrations of the Yangshan fluorite veins range from 0.606 to 3.405 ppm; the Nd concentrations range from 2.13 to 15.56 ppm; the Sm/Nd ratios range from 0.219 to 0.302, with an average value of 0.215; the ^147^Sm/^144^Nd ratios range from 0.1323 to 0.1827, with an average value of 0.16495; and the ^143^Nd/^144^Nd ratios range from 0.511887 to 0.511916, with an average value is 0.511902.

**Fig 3:**
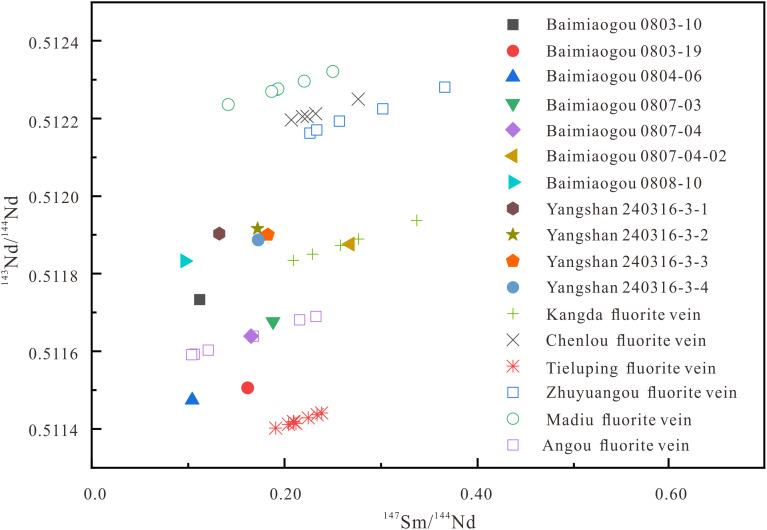
The ^147^Sm/^144^Nd-^143^Nd/^144^Nd correlation diagram of the Baimiaogou fluorite veinlets and Yangshan fluorite veins in the western Henan area, southern margin of the North China Craton.

The Baimiaogou fluorite veinlets have wide ranges of ^147^Sm/^144^Nd and ^143^Nd/^144^Nd ratios, with data points scattered widely and failing to define a single isochron with a consistent initial ^143^Nd/^144^Nd ratio. The compilation of fluorite veins in the western Henan region on the southern margin of the North China Craton yields mineralization ages ranging from 118 to 126 Ma, with most ages clustering at approximately 120 Ma. Using 120 Ma as the mineralization age for both the Baimiaogou veinlets and the Yangshan veins, we calculated the initial ^143^Nd/^144^Nd ratios, *ε*_Nd_(*t*) values, and two-stage model ages (*T*_DM2_). The initial ^143^Nd/^144^Nd ratios of 0.511379–0.511757, *ε*_Nd_(*t*) values of –21.55 to –14.17, and *T*_DM2_ ages of 1.64–1.93 Ga for the Baimiaogao samples are summarised in S1 Table. The Yangshan fluorite veins yield initial ^143^Nd/^144^Nd ratios of 0.511751–0.511799, *ε*_Nd_(*t*) values of –14.29 to –13.36, and *T*_DM2_ ages of 1.58–1.60 Ga. Regional fluorite veins exhibit initial ^143^Nd/^144^Nd ratios ranging from 0.511245 to 0.512125 and *ε*_Nd_(*t*) values ranging from –23.99 to –7.01 (S2 Table and Fig 3).

### C-O isotopes

**Fig 4:**
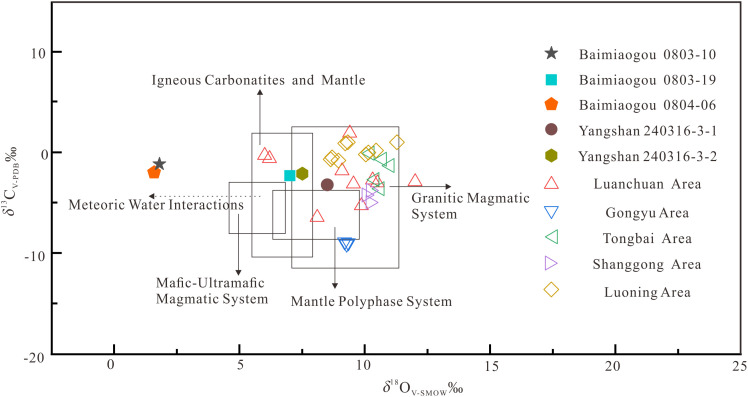
C-O isotope plot of calcite associated with fluorite in the western Henan area, southern margin of the North China Craton (base map according to the literature [43–45]). Data source: Baimiaogou and Yangshan areas from this study; other areas from the literature [35,45].

The *δ*^13^C_V-PDB_‰ values of calcite associated with fluorite veinlets in the Baimiaogou deposit range from –2.4 ‰ to –1.2 ‰, and the *δ*^18^O_V-SMOW_‰ values range from 1.6 ‰ to 7 ‰. The *δ*^13^C_V-PDB_‰ values of calcite in the Yangshan fluorite range from –2.2 ‰ and –3.3 ‰, and the *δ*^18^O_V-SMOW_‰ values range from 7.5 ‰ to 8.5 ‰ (S3 Table and Fig 4).

## Discussion

### Heterogeneity of fluorite material sources

By comparing the initial ^143^Nd/^144^Nd ratios and *ε*_Nd_(*t*) values of individual fluorite veins with those of potential source regions (i.e., sedimentary strata or magmatic rocks), the material sources of fluorite can be effectively traced [27,46,47]. For example, Graupner’s study on fluorites from various lithologies in the Vergenoeg fluorite deposit, South Africa, demonstrated that fluorites derived from a single source exhibit highly uniform initial Nd isotopic ratios, whereas variations in these ratios indicate the influx of exotic components [48].

Furthermore, comparing the initial^143^Nd/^144^Nd ratios and *ε*_Nd_(*t*) values among different veins allows for the identification of provenance variations [49–51]. For instance, the distinct initial ^143^Nd/^144^Nd ratios of the Ribeira fluorites in Paraná State, Brazil, suggest the involvement of two different types of source rocks [52]. Similarly, Peng [22] investigated fluorites from various locations within a single metallogenic belt in the Qinglong area, southwestern Guizhou Province, China. The results revealed that different ore bodies formed during the same metallogenic epoch display varying initial ^143^Nd/^144^Nd ratios, reflecting the contributions of multiple sources [22].

The Baimiaogou and Yangshan fluorite veinlets have contrasting Nd isotope characteristics, with *ε*_Nd_(*t*) values ranging from –21.55 to –13.36 and corresponding two-stage model ages (*T*_DM2_) ranging from 1.57 to 1.93 Ga. These isotopic features suggest that the ore-forming materials were influenced by both Yanshanian magmatic-hydrothermal fluids and the ancient continental crustal basement.

Some Baimiaogou fluorite veinlets (samples 0803−10, 0808−10, and 0807-04-02) and the Yangshan samples yield *ε*_Nd_(*t*) values (–16.36 to –13.36) that fall within the range of the Heyu pluton (–16.4 to –11.2), and their *T*_DM2_ ages (1.57 to 1.72 Ga) closely align with those of the Heyu pluton (1.6 to 2.1 Ga) [53]. Combined with the magmatic carbon signature of calcite in sample 0803−10, these findings indicate that the ore-forming materials in this group of fluorite are predominantly derived from magmatic-hydrothermal fluids exsolved from the Heyu pluton. Samples 0804−06 and 0803−19 exhibit extremely low *ε*_Nd_(*t*) values (–21.55 and –21.29, respectively) and ancient *T*_DM2_ ages (~1.90 Ga). Although *δ*^13^C_V-PDB_‰ values in associated calcite indicate a magmatic contribution, their Nd isotopes and *T*_DM2_ ages closely resemble those of the Tieluping fluorite vein (*ε*_Nd_(*t*) = –23.99), which occurs within the Taihua Group. This likely reflects the extraction of Palaeoproterozoic or even older metamorphic basement materials during magmatic fluid migration, imprinting ancient crustal information on the precipitated fluorite in terms of Nd isotopes and *T*_DM2_ model ages. Samples 0807−03 and 0807−04 have *ε*_Nd_(*t*) values of –19.01 and –18.62, respectively, with *T*_DM2_ ≈ 1.8 Ga, placing their Nd isotopic signatures between those of the Heyu pluton and the Taihua Group basement, similar to the Angou fluorite vein (*ε*_Nd_(*t*) = –19.02), whose rare earth elements are derived mainly from the host wall rock (Taihua Group gneiss). This suggests that during mineralization, magmatic fluids underwent some degree of material exchange with the Taihua Group, resulting in Nd isotopic signatures recording different end-member components. Such mixing of different end-members causing deviations in Nd isotopes is supported by the mass balance equation of DePaolo [49]:


$$\begin{array}{c} \epsilon_{𝐍𝐝}^{𝐦}=\frac{{𝐗}^{\alpha }{[}^{144}𝐍𝐝{]}^{\alpha }\epsilon_{𝐍𝐝}^{\alpha }+\left(1-{𝐗}^{\alpha }\right){[}^{144}𝐍𝐝{]}^{\beta }\epsilon_{𝐍𝐝}^{\beta }}{{𝐗}^{\alpha }{[}^{144}𝐍𝐝{]}^{\alpha }+\left(1-{𝐗}^{\alpha }\right){[}^{144}𝐍𝐝{]}^{\beta }} \end{array}$$

where m denotes the mixture, X^α^ and 1-X^α^ represent the mass fractions of Nd from the two end-members in the mixed system, [^144^Nd]^α^ and [^144^Nd]^β^ are the molar concentrations of ^144^Nd in the two end-members (approximated by Nd weight concentrations), and *ε*_Nd_^α^ and *ε*_Nd_^β^ are the *ε*_Nd_(*t*) values of the two end-members. Taking end-member A as the initial magmatic-hydrothermal fluid evolved from the Heyu pluton (represented by some Baimiaogou and Yangshan samples, with *ε*_Nd_(*t*) taken as –15.0) and end-member B as the ancient Taihua Group metamorphic basement (using the Tieluping ore-hosting wall rock as a reference, with *ε*_Nd_(*t*) taken as –24.0), calculations show that when end-member B contributes 40–45% to the mixture, the resulting *ε*_Nd_(*t*) values fall between –19.5 and –18.6. This quantitative mixing range closely matches the measured values of samples 0807−03 and 0807−04 (–19.01 and –18.62, respectively), indicating that magmatic fluids may have extracted variable proportions of ancient continental crustal materials during migration and precipitation, causing variations in initial Nd isotope ratios among the fluorite veinlets.

These Nd isotope characteristics are further supported by previous provenance studies on different fluorite veins in the region. The Chenlou and Zhuyuangou fluorite veins have *ε*_Nd_(*t*) values of –8.8 and –9.7, respectively, reflecting a greater contribution from juvenile crust or mantle-derived materials [31–32]. The Kangda fluorite vein yields an *ε*_Nd_(*t*) value of –15.9, indicating a relatively uniform source supplied by the Heyu pluton [39]. The Tieluping fluorite vein has an *ε*_Nd_(*t*) value of –23.99 and occurs mainly within the ancient Taihua Group basement, where wallrock influence dominated the mineralization [14].

Carbon and oxygen isotope values vary systematically among different source reservoirs [43,54,55], making C-O isotopes useful for tracing ore-fluid sources and evolution. Associated calcite in the Baimiaogou fluorite veinlets has relatively uniform *δ*¹³C_V-PDB_‰ values (–2.4‰ to –1.2‰), which are comparable to those of mantle or igneous rocks (–5 ± 2) [56] and their released CO_2_ (–2 to –8) [57], and significantly higher than those of continental crust (–7), atmospheric CO_2_ (ca. –8) [58], or dissolved CO_2_ in freshwater (–20 to –9) [56]. These findings indicate that the carbon in calcite and fluids within the Baimiaogou fluorite veinlets derives from a magmatic source (Fig 4), which is consistent with previous conclusions that Yanshanian magmatism supplied material sources for regional fluorite mineralization [59].

In contrast, the associated calcite in the Baimiaogou fluorite veinlets shows a wide range of *δ*^18^O_V-SMOW_‰ values (1.6–7.0‰), with a pronounced “oxygen isotope shift”. Significant changes in the oxygen isotopic composition of mantle/magmatic source materials typically require the substantial addition of high- or low-*δ*^18^O_V-SMOW_‰ materials to hydrothermal fluid [60]. These findings suggest that the ore-forming fluids in these veinlets originated as magmatic-hydrothermal fluids exsolved from a closed magmatic system, retaining some original compositional characteristics during ascent, but later evolved under the influence of an open system [61], leading to the influx of significant amounts of low-*δ*^18^O_V-SMOW_‰ meteoric water. This caused a negative shift in *δ*^18^O_V-SMOW_‰ values and resulted in isotopic heterogeneity among different fluorite veinlets, indicating that multiple sources with different characteristics contributed to the ore-forming fluids in the Baimiaogou veinlets.

Compared with the Baimiaogou veinlets, the Yangshan fluorite veins contain associated calcite with relatively uniform *δ*^13^C_V-PDB_‰ values (–3.3‰ and –2.2‰) and *δ*^18^O_V-SMOW_‰ values (7.5 ‰and 8.5‰), confirming that the carbon in the ore-forming fluids originated from deep magmatic sources. However, their *δ*^18^O_V-SMOW_‰ values are significantly higher than those of Baimiaogou and fall within the typical range of those of primary magmatic water (5–10‰). The absence of an “oxygen isotope shift” in the Yangshan veins suggests that during calcite and fluorite precipitation, the system remained relatively closed, or that precipitation occurred at an early stage of mineralization without significant meteoric water incursion. These findings indicate that while Yangshan and Baimiaogou likely formed under the same mineralization setting with consistent material sources, the Yangshan veins preserved their original magmatic-hydrothermal fluid signatures.

Regional compilation of C-O isotope data from calcite in fluorite deposits in the western Henan region on the southern margin of the North China Craton (Fig 4) reveals that *δ*^13^C_V-PDB_‰ and *δ*^18^O_V-SMOW_‰ values are highly concentrated within individual veins but significantly differ among different veins, all of which are closely related to magmatic-hydrothermal systems. This pattern reflects the evolutionary characteristics of regional ore-forming fluids: isotopic homogeneity within a single vein suggests relatively stable fluid properties within local structural fractures, resulting from rapid fluid filling and precipitation during a single, short-lived event; isotopic heterogeneity among different veins implies local heterogeneity in fluid evolution processes within different fault systems or ore-hosting spaces.

### Controlling factors

#### Magmatic factors.

Magmatic activity affects the initial ^143^Nd/^144^Nd ratios and *ε*_Nd_(*t*) values of minerals/rock bodies through multiple mechanisms: (1) Source region. During melt differentiation events such as partial melting or magmatic fractionation, Sm (a middle rare earth element) tends to remain in the residual phase, whereas Nd (a light rare earth element) is more readily incorporated into the new melt phase. This results in different Sm/Nd ratios in magmas derived from distinct sources or having undergone different degrees of differentiation. Consequently, these magmas possess different initial ^143^Nd/^144^Nd ratios even before ^147^Sm decay [23,49]. For example, variations in the initial ^143^Nd/^144^Nd ratios of fluorite formed from magmatic sources along the western margin of the Bohemian Massif in Germany confirm inherent Nd isotope heterogeneity within the regional magma source [51]. Similarly, highly variable initial Nd isotopic compositions recorded in plutons from several areas in northwestern Canada reflect magmatic differentiation events [17]. (2) Contamination. During emplacement, interactions between magma and crustal material can alter the magma’s initial ^143^Nd/^144^Nd ratio of the magma [62,63]. Significant internal Nd isotope variations within plutons in western Shandong, China [64,65] and in several areas of the western United States [66] have been attributed to magma-wall rock/strata interactions.(3) Magmatic-Hydrothermal alteration. Hydrothermal activity associated with magmatism can cause differential mobilization and redistribution of Sm and Nd from the protolith. This process disturbs the Sm/Nd ratio and consequently the initial ^143^Nd/^144^Nd ratio of the system [67,68]. Variations in Nd isotopic compositions of granites from southeastern France [69] and Australia [70] correlate with varying degrees of hydrothermal alteration, which also led to changes in the ^143^Nd/^144^Nd ratios of associated fluorite.(4) Mixing of fluids from multiple sources. The mixing of fluid end-members derived from different sources in varying proportions can produce differences in initial ^143^Nd/^144^Nd ratios [71,72]. Regional faulting in central-western Argentina facilitated the mixing of granitic magmatic-hydrothermal fluids with meteoric water, resulting in perturbations in the ^143^Nd/^144^Nd ratios of fluorite [67].

As discussed earlier, the variations in the initial ^143^Nd/^144^Ndratios of fluorite veins in the western Henan region on the southern margin of the North China Craton reflect material sources primarily related to regional granitic magmas [73–77]. Therefore, analysing the source characteristics and evolutionary history of these regional granites is key to understanding the multiple material sources of fluorite in this area.

From the Middle Triassic to the Early-Middle Jurassic, the subduction and collision of the Yangtze Craton beneath the North China Craton [78] led to the compressional thrusting of the South Qinling crust into the southern margin of the North China Craton in the western Henan region, resulting in significant crustal thickening.During the Jurassic-Cretaceous transition, the regional tectonic regime underwent a significant shift from predominantly compression to extension [79–82].This shift triggered the large-scale upwelling of asthenospheric material. This process led to significant lithospheric thinning and underplating,which in turn baked the overthickened ancient lower crust of the North China Craton, inducing extensive partial melting and thereby generating intense regional magmatism. Consequently, most researchers have proposed that the material source of granites in the western Henan region on the southern margin of the North China Craton was primarily derived from the crystalline basement of the South Qinling Belt, with additional contributions from the Taihua Group and Xiong’er Group of the North China Craton, as well as mantle-derived components [83–89].

This multisource nature of the magmas implies that the Yanshanian granitic batholiths in the western Henan region had heterogeneous initial ^143^Nd/^144^Nd ratios and *ε*_Nd_(*t*) values at the time of their formation (S4 Table). The hydrothermal fluids exsolved from these granites preserved these initial isotopic heterogeneities, thereby providing the foundation for the formation of fluorite deposits with varying initial ^143^Nd/^144^Nd ratios and *ε*_Nd_(*t*) values.

**Fig 5:**
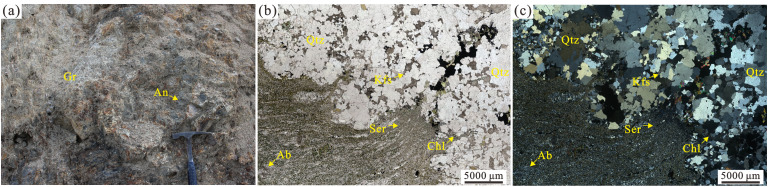
Contamination and alteration of granite and andesite in Baimiaogou area and thewestern Henan area, southern margin of North China Craton. (a)-Contamination features between granite and andesite, predominantly granitic overall, showing altered andesitic xenoliths of varying sizes and irregular shapes; (b)-microscopic characteristics of granite and andesite contamination and granite alteration (-). The andesite exhibits intense sericitization and intrudes into the granite along fractures. The potassium and silicification characteristics of plagioclase in granite are obvious. Sample number0804−14; (c)-same view as (b)-under crossed polars (+).

During emplacement, regional granitic intrusions commonly undergo assimilation and contamination with country rocks. In Baimiaogou, significant contamination occurs at the contact between the Heyu granite and andesites of the Xiong’er Group. Additionally, the Heyu granite frequently exhibits hydrothermal alteration, such as K-feldspar alteration and silicification (Fig 5). Consequently, even fluids exsolved from the same granitic batch within the same region may display variations in their initial ^143^Nd/^144^Nd ratios because of differences in the extent of contamination and alteration processes [66,72].

The ore-controlling fault structures in the Baimiaogou fluorite veinlets are brittle in nature, which allows meteoric water to readily infiltrate and mix with the ore-forming hydrothermal fluids in varying proportions. This mixing process also contributes to the variation in initial^143^Nd/^144^Nd ratios. Similar processes have been documented in fluorite deposits in other regions, including Sardinia, Italy [90], Córdoba, Argentina [67], and southeastern Sichuan, China [91].

Fig 5a was obtained through onsite photography by the author, while Fig 5b and 5c were captured via microscopy photography based on actual collected samples; no copyrighted material was used.

#### Structural control factor.

Although the fluorite veins (veinlets) in the western Henan region on the southern margin of the North China Craton exhibit multisource characteristics, the fluorite of the same individual vein within the same area displays similar initial ^143^Nd/^144^Nd ratios and *ε*_Nd_(*t*) values, which may be related to the fluctuating nature of tectonic activity [92–94].

Faults generated by tectonic events typically serve as both conduits for fluid migration and spaces for ore precipitation. The connectivity of these fault systems determines whether the ore-forming fluids were derived from a single source and evolved within a closed system, or if fluids from multiple sources converged and mixed. The initial ^143^Nd/^144^Nd ratios of fluorite are sensitive to this process [95–97]. Previous studies have attributed variations in ^143^Nd/^144^Nd ratios to several factors such as multisource fluid mixing and later hydrothermal disturbances, offering an important perspective for understanding Nd isotope differences among veins. However, these factors themselves may be controlled by deeper tectonic processes. The differences in the sources of different fluorite deposits in the Asturias region of Spain are due to the varying degrees of interaction between fluids and different lithologies (such as granite and basement rocks) or the lengths of flow paths. This difference is attributed to the variations in the fracture properties (connectivity, activity periods and structural characteristics) of the same fracture system [98,99]. This model is similar to those of other classic fluorite districts in Europe, such as the North Pennine Orefield and the Chaillac deposit [100,101]. Similarly, Xu Zhangzhang [102] reported that the ore-forming temperatures and trace element contents of fluorite veins in the Wuyi area, Zhejiang, China, vary with the nature of the controlling fault structures. In the Yixian area, Liaoning, China [103], the characteristics of the ore-controlling structures directly influence the scale, quality, and material sources of different fluorite veins.

The fluorite veins in the western Henan region on the southern margin of the North China Craton are strictly controlled by regional fault systems, primarily trending NW or NE, with deposits aligning in a bead-like distribution along these fault sets [11,40,104]. The regional NW-trending faults (the Luanchuan and Machaoying faults) have undergone long-term activity and control the tectonic evolution of the study area.Since the Mesozoic, these faults have been dominated by sinistral strike-slip motion, with late-stage extensional components [105,106]. NE-trending faults formed mainly during the late Mesozoic and exhibit transtensional characteristics [107–109].Ore-hosting structures of fluorite deposits in the western Henan region on the southern margin of the North China Craton typically develop at locations where the attitude of these fault sets changes [34,104], in secondary tensional fractures [31], and at the intersections of the two fault sets [33]. The fluorite deposits are often notably thick. For instance, the Chenlou fluorite vein ranges from 5.54 to 11.22 m in thickness, reaching a maximum of 21 m [9]; the Zhuyuangou vein varies from 1.00 to 16.81 m, averaging 3.02 m [33]; and the Kangda vein is 1.0 to 4.5 m thick [54]. These thick fluorite orebodies have strong continuity along both the strike and dip directions.

The Yangshan fluorite veins investigated in this study are controlled by regional NW-trending faults (striking 295–310°). Fluorite veins locally form at locations with variations in strike or dip. The phenomenon of fluorite cementing brecciated granite is observable, highlighting the characteristics of an ore-hosting structure.

Controlled by the same fault zone, the Baimiaogou fluorite veinlets are generally <20 cm in width. They are characterised by fracture swarms with diverse strikes (NW, NE, N-S, and E-W) and steep dips (70°–87°), with flower structures observable in cross-sections. Fluorite within these veinlets exhibits distinct variations in colour and crystallinity, indicating multiphase and pulsatory infiltration characteristics (Fig 2). These fractures likely served as migration pathways (i.e., ore-conducting structures) for the ore-forming hydrothermal fluids. Structural fractures with different attitudes are interpreted as secondary fractures of the main NW-trending strike-slip fault. The infilling veinlets represent syntectonic hydrothermal products formed during different pulsatory stages of faulting. Notably, they were influenced by fluids with distinct material sources or evolutionary pathways, even within a single veinlet.

During fault-controlled fluid flow, recurrent tectonic activity dynamically alters migration pathways, causing multiple fluid “batches/pulses” to migrate along different routes [50,110,111]. As hydrothermal fluids migrate, they gradually equilibrate with the wall rocks via dissolution-precipitation and exchange reactions. The Nd isotopic composition of the fluid depends on the elemental concentrations in the fluid, the Nd concentrations of various minerals in the wall rocks, the reaction kinetics between the fluid and mineral phases along the flow path, and the mixing efficiency within the fluid. Consequently, even for vein-type fluorite formed contemporaneously, structural variations on a relatively small scale can lead to distinct isotopic signatures in the fluids. This results in a wide range of initial ^143^Nd/^144^Nd ratios, which is a common characteristic of fractured hydrothermal systems [112,113]. This model of tectonic events driving pulsatory fluid evolution and influencing isotopic compositions is validated by studies on epithermal vein swarms in the Taemas district, New South Wales, Australia. In that district, the ^147^Sm-^143^Nd, *δ*^13^C_**V-PDB**_**‰**, and *δ*^18^O_**V-SMOW**_**‰** isotopic compositions of calcite and paragenetic fluorite within the same vein swarm exhibit significant variations, reflecting the diversity of fluid sources, migration pathways, and degrees of water-rock interaction in fractured hydrothermal systems [49]. Therefore, the nature and activity of the ore-conducting structures caused the Baimiaogou fluorite veinlets to possess varying initial ^143^Nd/^144^Nd ratios, *ε*_Nd_(*t*) values, and C-O isotopic compositions.

In contrast, relatively thick Yangshan fluorite veins are hosted within dilatational spaces formed by regional attitude variations, predominantly along NW-trending strike-slip faults. Within these veins, the fluorite exhibits largely uniform occurrences, colours, and textural-structural features, yielding consistent initial ^143^Nd/^144^Nd ratios. Furthermore, the C-O isotopic compositions of paragenetic calcite within the fluorite veins are generally identical (Fig 4). These findings indicate that the hydrothermal fluids intruding into these spaces share identical material sources and evolutionary pathways, suggesting that they are the products of a single tectonic pulsatory event. Other fluorite veins in the Yuxi region on the southern margin of the North China Craton are similarly hosted within distinct ore-hosting spaces, and each individually yields a relatively uniform initial ^143^Nd/^144^Nd ratio. This implies that, similar to the Yangshan fluorite veins, these regional veins were also individual products of single tectonic pulsatory events. On a regional scale, however, significant discrepancies exist in the initial ^143^Nd/^144^Nd ratios among different fluorite veins, demonstrating that these distinct veins were generated during different stages of tectonic pulsation.

## Conclusions

(1) Fluorite mineralization in the western Henan region derives mainly from Yanshanian magmatic-hydrothermal fluids and contributions from the ancient continental crustal basement. The samples yield *ε*_Nd_(*t*) values ranging from –21.55 to –13.36 and *T*_DM2_ model ages ranging from 1.57 to 1.93 Ga, indicating the involvement of multiple material sources. Associated calcite has *δ*^13^C_V-PDB_‰ values of –3.3‰ to –1.2‰7.0, confirming that the initial carbon originated from deep-seated magmatic fluids exsolved from the Heyu pluton. Isotopic mass balance modelling suggests that magmatic-hydrothermal fluids were extracted and mixed with materials from the ancient Taihua Group metamorphic basement during migration, causing variations in initial ^143^Nd/^144^Nd ratios in the fluorite.
(2) Ore-forming fluid evolution in different structural settings significantly differs between closed and open systems. The oxygen isotopic signature of primary magmatic water is preserved in the Yangshan fluorite veins (*δ*^18^O_V-SMOW_‰ = 7.5–8.5‰), suggesting that the fluid system remained relatively closed during precipitation or that mineralization occurred at an early stage. In contrast, the Baimiaogou fluorite veinlets display a pronounced oxygen isotope shift (*δ*^18^O_V-SMOW_‰ values as low as 1.6–7.0‰), reflecting an open structural system with significant incursion of meteoric water during the late stage of fluid evolution.
(3) Isotopic heterogeneity within and among fluorite deposits results from the coupling between magmatic evolution and tectonic activity. Assimilation and contamination of wall rocks (e.g., the Taihua Group and Xiong’er Group andesites) during magma emplacement, together with hydrothermal alteration, disturbed the Nd isotopic composition of the fluids. The nature of fault structures controls the degree of fluid homogenization. As conduit structures, the Baimiaogou veinlets experienced multiple tectonic pulses with dynamically changing fluid flow paths, leading to small-scale isotopic heterogeneity within individual veinlets. In contrast, the thick Yangshan fluorite veins, which represent host structures, formed during a single large-scale tectonic pulse, allowing prolonged fluid evolution and thorough mixing within a relatively stable space, resulting in highly homogeneous isotopic signatures.

## Supporting information

S1 Table Sm-Nd isotope data for fluorite from the Baimiaogou fluorite veinlets and the Yangshan fluorite veins in the southern margin of the North China Craton. (XLSX)

S2 Table Initial ^143^Nd/^144^Nd ratios and *ε*_Nd_(*t*) values of fluorite veins in the western Henan region, southern margin of the North China Craton. (XLSX)

S3 Table C-O isotope data for calcite in the Baimiaogou fluorite veinlets and Yangshan fluorite vein in the western Henan region, southern margin of the North China Craton. (XLSX)

S4 Table Initial ^143^Nd/^144^Nd and *ε*_Nd_(*t*) values of granites and strata in different areas of western Henan, southern margin of the North China Craton. (XLSX)
